# Neuroglobin-overexpression reduces traumatic brain lesion size in mice

**DOI:** 10.1186/1471-2202-13-67

**Published:** 2012-06-15

**Authors:** Song Zhao, Zhanyang Yu, Gang Zhao, Changhong Xing, Kazuhide Hayakawa, Michael J Whalen, Josephine M Lok, Eng H Lo, Xiaoying Wang

**Affiliations:** 1 Departments of Orthopedic and Neurosurgery, The First Bethune Hospital of Jilin University, Changchun, Jilin, China; 2 Neuroprotection Research Laboratory, Departments of Neurology and Radiology, Massachusetts General Hospital, Harvard Medical School, Charlestown, MA, USA; 3 Department of Pediatrics, Pediatric Critical Care Medicine, Massachusetts General Hospital, Harvard Medical School, Charlestown, MA, USA; 4 Neuroprotection Research Laboratory, Departments of Neurology and Radiology, Massachusetts General Hospital, 149 13th Street, Room 2401, Charlestown, MA, 02129, USA

**Keywords:** Neuroglobin, Neuroprotection, Controlled cortical impact, Oxidative stress, Traumatic brain injury

## Abstract

**Background:**

Accumulating evidence has demonstrated that over-expression of Neuroglobin (Ngb) is neuroprotective against hypoxic/ischemic brain injuries. In this study we tested the neuroprotective effects of Ngb over-expression against traumatic brain injury (TBI) in mice.

**Results:**

Both Ngb over-expression transgenic (Ngb-Tg) and wild-type (WT) control mice were subjected to TBI induced by a controlled cortical impact (CCI) device. TBI significantly increased Ngb expression in the brains of both WT and Ngb-Tg mice, but Ngb-Tg mice had significantly higher Ngb protein levels at the pre-injury baseline and post-TBI. Production of oxidative tissue damage biomarker 3NT in the brain was significantly reduced in Ngb-Tg mice compared to WT controls at 6 hours after TBI. The traumatic brain lesion volume was significantly reduced in Ngb Tg mice compared to WT mice at 3 weeks after TBI; however, there were no significant differences in the recovery of sensorimotor and spatial memory functional deficits between Ngb-Tg and WT control mice for up to 3 weeks after TBI.

**Conclusion:**

Ngb over-expression reduced traumatic lesion volume, which might partially be achieved by decreasing oxidative stress.

## Background

Neuroglobin (Ngb) is a member of the tissue globin family with a high affinity for oxygen, and it is widely expressed in the neurons of vertebrate central and peripheral nervous systems, retina, and endocrine tissues [1-3]. Accumulating evidence from our lab and others has demonstrated that Ngb over-expression is neuroprotective against hypoxic/ischemic brain injuries in cell cultures and stroke animal models, as well as in beta-amyloid-induced neurotoxicity in vitro and Alzheimer’s phenotype in mice [4-7]. Although the underlying neuroprotective mechanisms remain largely unknown, experimental studies have suggested that Ngb may act as an oxygen sensor in regulating the signaling pathway, attenuating oxidative stress and preserving mitochondrial function [4,8-11]. Traumatic brain injury is one of the major acute neurological disorders, but the effects of Ngb over-expression on its neurological outcomes remain uninvestigated. Therefore, in this study we tested whether Ngb over-expression is neuroprotective against traumatic brain injury in mice.

## Methods

All animal experiments were performed following protocols approved by the Massachusetts General Hospital Animal Care and Use Committee in compliance with the National Institutes of Health Guide for the Care and Use of Laboratory Animals.

### Controlled cortical impact model of traumatic brain injury

The standard controlled cortical impact model of traumatic brain injury was performed as previously described [12,13]. Briefly, a right craniotomy was made using a portable drill and 5 mm trephine over the right parieto-temporal cortex, and the bone flap was removed. Mice were subjected to CCI using a pneumatic cylinder with a 3-mm flat-tip impounder, velocity 6 m/s, depth of 0.6 mm, and 150 ms impact duration. Ngb-Tg mice and WT control mice in C57BL/6 background were ear-tagged and assigned randomly into two equal-sized groups, blinded to the investigators.

### Immunohistochemistry

Immunohistochemistry was performed following the standard method as we previously described [5]. Briefly, at 6 h after TBI, mice were anesthetized with an overdose isoflurane and were transcardially perfused with chilled (4°C) PBS (pH 7.4). Coronal sections (12 μm) on slides were fixed in 100% cold aceton and blocked in PBS with 3% bovine serum albumin for 1 h and incubated at 4°C overnight in a PBS solution containing the rabbit anti-Ngb antibody (1:50; Santa Cruz Biotechnology, CA, US) in 0.3% bovine serum albumin. The sections were then washed and incubated for 1 h with Rhodamine (TRITC)-AffiniPure Goat Anti-Rabbit IgG (H + L)(1:200;Jackson ImmunoResearch, PA, US). Signals in peri-lesion cortex were examined using an Olympus microscope (BX51; Olympus) equipped with rhodamine filter set.

### Western blots

Western blots analysis was performed following the standard method as we previously described [5]. Briefly, injured brain hemisphere tissues were dissected on ice, followed by protein extraction. Protein concentrations in the supernatants were measured with Bradford assay (Bio-Rad, Hercules, CA, USA). Equal amount of protein were separated in a 4-20% Tris-glycine gel (Invitrogen) (40 μg/lane) and then transferred onto PVDF Membranes. The membranes were incubated with primary anti-Ngb antibody (1:1000; Biovendor R&D, NC, USA) and anti-β-actin antibody (1:10000; Sigma) at 4°C overnight. Then the membranes were incubated with horseradish peroxidase-linked rabbit polyclonal anti-Chicken IgY (1:2000, Abcam, MA, USA) and anti-mouse IgG (1:10000, GE Healthcare, UK), respectively for 1 hr at room temperature, developed by enhanced chemiluminescent (Pierce, Rockford, IL, USA). Densitometric analysis was performed for quantitation with Image J software.

### Slot-immunoblotting analysis

3-nitrotyrosine (3NT) production in brain tissues after TBI was assessed with slot-immunoblotting analysis followed by a standard method described by others, with minor modifications [14,15]. Briefly, at 6 h after TBI, mice were transcardially perfused with chilled (4°C) PBS (pH 7.4), and the brains were carefully removed and placed in a cooled (6°C) brain matrix. A 3 mm thick slice was cut in coronal orientation and separated in quadrants. The right-upper (traumatized) region was collected, frozen in liquid nitrogen and stored at -80°C until further processing. To measure 3-nitrotyrosine (3NT) production, an aliquot of each ipsilateral brain sample (1 μg) was diluted with 200 μl of tris-buffered saline (TBS) and transferred to a protran nitrocellulose membrane (Whatman Inc., ME, US) by a Minifold I vacuum slot blot apparatus (Whatman Inc., ME, US). After the samples were loaded into the slots, they were allowed to filter through the membrane by vacuum. Each slot was then washed with 200 μl TBS, which was allowed to filter through the membrane again. The membranes were disassembled from the apparatus and incubated in a TBS blocking solution with 5% milk for 1 h at room temperature. Then the membranes were incubated with rabbit polyclonal anti-nitrotyrosine antibody (1:2000; Sigma, MA, US) at 4°C overnight. After the primary incubation, the membranes were washed and then incubated again with horseradish peroxidase-linked anti-rabbit IgG (1:2000, Abcam, MA, USA) for 1 h at room temperature, developed by enhanced chemiluminescent (Pierce, Rockford, IL, US). Densitometric analysis was performed for quantitation with Image J software.

### Assessments of sensorimotor functions

A 10-point neurological severity score (NSS) was used for the assessment of posttraumatic neurological impairment, as previously described [16,17]. The score consists of 10 individual clinical parameters, including tasks on motor function, alertness, and physiological behavior, whereby 1 point is given for failure of one task and no points are given for success. A maximal NSS of 10 points indicates severe neurological dysfunction, with failure at all tasks. The NSS was assessed at 1, 3, 5, 7, 10, 14 and 21 days after TBI. All mice were trained and pretested prior to injury. Vestibulomotor function was assessed using a standard wire-grip test [18]. Mice were placed on a metal wire (45 cm long) suspended 45 cm above a foam pad and were allowed to traverse the wire for 60 seconds. The latency that a mouse remained on the wire within a 60 second interval was measured, and wire grip scores were quantitated using a 5-point scale. 0 = fell off the wire within the 30 s period, 1 = held on in some way for 30 s, 2 = held on with four paws for ≥5 s, 3 = held on with four paws and placed tail on wire for ≥5 s, 4 = held on with four and move to the end of the wire, 5=move to the end of the wire and go down to the ground with four paws The tests were performed in triplicate, and an average value was calculated for each mouse on each day of testing. Baseline assessment was done the day before injury.

### Assessments of spatial memory functions

The Morris Water Maze task was used to evaluate spatial memory performance from 15 days to 21 days after TBI as previously described [18]. Briefly, performance in the MWM was quantitated by latency to the platform. The apparatus consisted of a white pool (90 cm diameter, 60 cm deep) filled with water to 29 cm depth, with several highly visible cues located on the walls of each of the four quadrants. Water temperature was maintained between 21°C and 25°C. A clear Plexiglass goal platform 5 cm in diameter was positioned 0.5 cm below the water surface approximately 15 cm from the southwest wall. Each mouse was subjected to a series of four trials per day. For each trial, mice were randomized to one of four starting locations (north, south, east, or west) and placed in the pool facing the wall. Mice were given a maximum of 60 seconds to find the submerged platform. If the mouse failed to reach the platform by 60 seconds, it was placed on the platform by the experimenter and allowed to remain there for 15 seconds. Mice were placed in a warming chamber for at least 4 minutes between trials. To control for possible differences in visual acuity or sensorimotor function between groups, two trials were performed using a visible platform raised 1 cm above the surface of the water. Performance in the MWM was quantitated by latency to find the platform.

### Quantitation of traumatic lesion volume

Quantitation of traumatic lesion volume was performed by following a standard method as previously described [5]. At 3 weeks after TBI, mice were sacrificed and transcardially perfused with chilled (4°C) PBS, pH7.4, followed by 4% paraformaldehyde in 0.1 M PBS. Frozen brains were sectioned with microtome into 20-μm-thick coronal slices. Every 20th section was mounted onto a glass slide and stained with hematoxylin and eosin. Histological lesion areas were quantified with a standard computer-assisted image analysis program (Image J), and the lesion areas were then integrated to obtain total lesion volumes in cubic millimeters.

### Statistical analysis

All data were expressed as mean ± SD. Sensorimotor and MWM were analyzed by two-factor repeated-measures analysis of variance (group × time). Slot- immunobloting and western blot data were analyzed by one-way ANOVA. Lesion volumes were analyzed by t-test. For all comparisons, P < 0.05 was considered significant.

## Results and discussion

Increasing evidence has demonstrated that Ngb plays an important role in neuroprotection against hypoxic/ischemic brain injury, such as stroke and other related neurological disorders [5,19,20]. Our previous studies showed that Ngb overexpression reduces tissue infarction and markers of oxidative stress in a focal stroke mouse model [5]. Recent reports by others have shown that Ngb gene expression was increased after TBI, but its effects on outcomes were not examined [21,22]. In this short study we tested our hypothesis that Ngb over-expression may protect against traumatic brain injury in mice. We performed three sets of experiments to examine (1) changes in brain Ngb protein expression after TBI, (2) the effects of Ngb over-expression in a mechanistic endpoint-oxidative tissue damage, and (3) the neurological outcomes of neurobehavioral deficits and traumatic lesion size at three weeks after TBI. Both Ngb-Tg and WT control mice were examined and compared.

**Figure 1:**
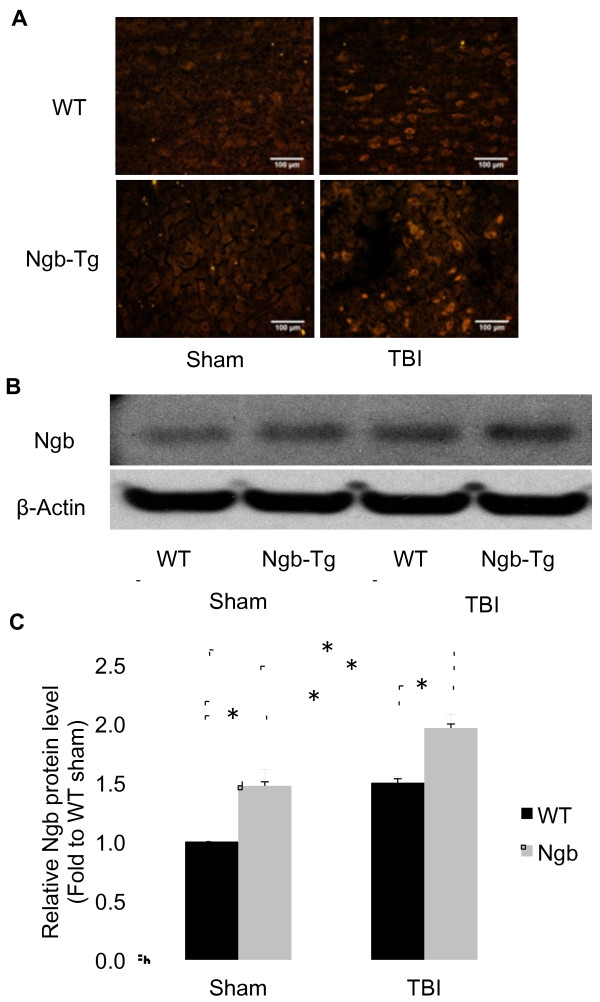
**Ngb expression levels in mouse brains after TBI.A**. Representative immunohistochemistry of Ngb protein expression in WT and Ngb-Tg mice cortex of peri-lesion area at sham animals and at 6 h after TBI. Original magnification × 200 in all photomicrographs. **B**. Representative western blot of Ngb protein expression in WT and Ngb-Tg mice cortex of peri-lesion area at sham animals and at 6 h after TBI, β-actin served as equal protein loading controls. **C**. Quantitation of Ngb protein levels. Data were expressed as mean ± SD. *P < 0.05, n = 5 per group.

In the first set of experiments, we found that Ngb protein level in the injured brains were significantly increased in both Ngb-Tg and WT mice compared to their sham controls at 6 h after TBI. This examination by immunohistochemistry (Figure 1A) and western blot analysis (Figure 1B) was consistent with a report that showed a transient increase of brain Ngb expression with peak at 6 h after TBI in rats. That study also showed Ngb over-expression decreased mechanical injury-induced neuron death [23]. In our present study, quantification of western blot analysis showed that the basal levels of Ngb protein expression (149% of WT sham control mice) and the CCI-induced increase of Ngb protein levels at 6 h after TBI (196% of WT sham control mice) were significantly higher in Ngb-Tg mouse brains compared to WT TBI controls (147% of WT sham control mice) (Figure1C). While this difference suggested that TBI induces Ngb protein expression, the basal and TBI-induced Ngb protein levels were still significantly higher in Ngb-Tg mouse brains, validating the possible causality between the different brain Ngb protein levels in association with different neurological outcomes after TBI of Ngb-Tg and WT control mice.

**Figure 2:**
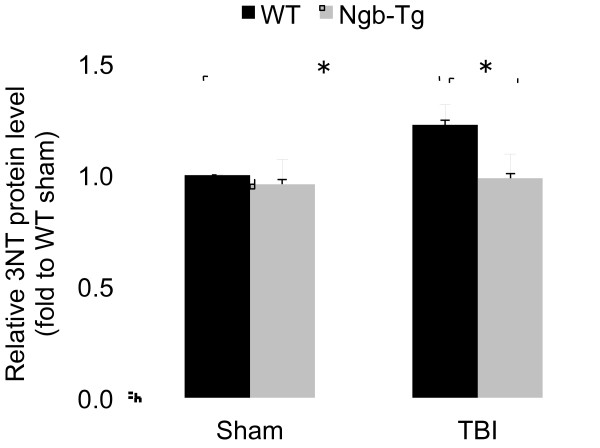
**Relative 3NT protein levels. Brian samples were collected at 6 h postinjury in sham and TBI-injured WT and Ngb-Tg mice.** Slot-immunoblotting analysis was used to determine relative 3NT protein levels. Data were expressed as mean ± SD. *P < 0.05, n = 5 per group.

Previous studies have demonstrated that Ngb over-expression can reduce hypoxia/ischemia-induced oxidative cell damage in cultured neurons and focal cerebral ischemia in mice [5,19]. In the second set of experiments, we tested and compared a common oxidative tissue damage biomarker, 3NT production, in TBI-injured brains of Ngb-Tg and WT control mice. We found that there was a significant reduction of 3NT level at 6 h after TBI in Ngb-Tg mouse brains as compared to WT controls (Figure 2), indicating TBI-induced oxidative tissue damage may be diminished by Ngb over-expression.

**Figure 3:**
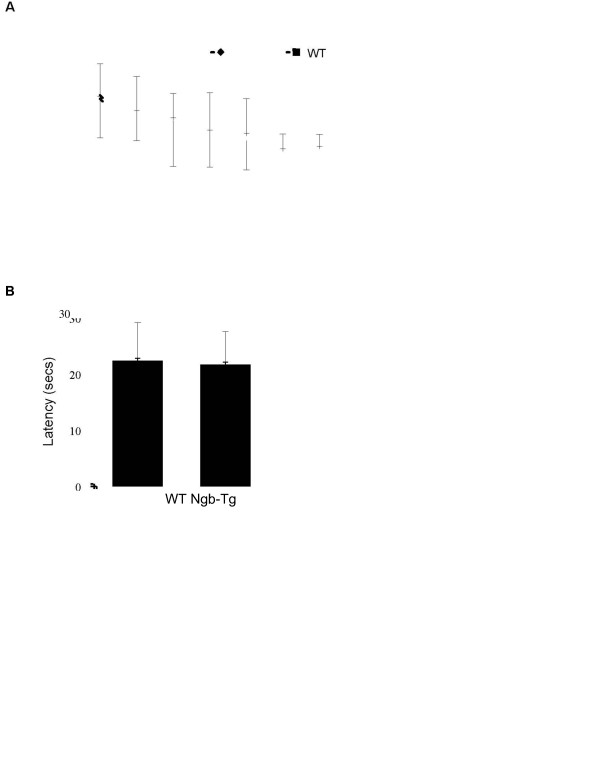
**Spatial memory acquisitions in WT and Ngb-Tg mice after TBI.A**. Morris water maze latencies were measured on days 15-21 for hidden and visible platform trials after TBI. **B**. Morris water maze latencies were measured on days 15-21 for probe trials after TBI. Data were expressed as mean ± SD. n = 15 for WT and 11 for Ngb-Tg per group, respectively.

**Figure 4:**
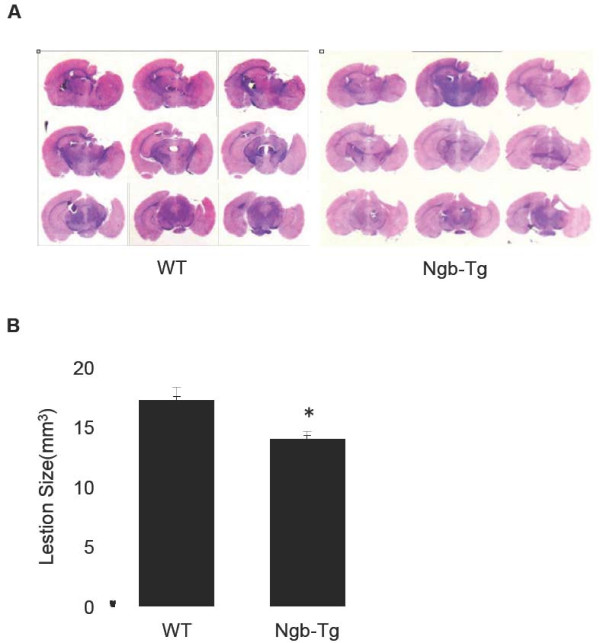
**Measurements of cortical lesion volume in WT and Ngb-Tg mice.A**. Representative photomicrographs of the traumatic lesions in H&E stained WT and Ngb-Tg mouse brain sections at 21 days after TBI. **B**. Traumatic brain lesion size. Data were expressed as mean ± SD. *P < 0.05, n = 15 for WT and 11 for Ngb-Tg per group, respectively.

In the last set of experiments, we examined neurological outcomes of neurobehavioral deficits for up to three weeks after TBI and traumatic brain lesion size at three weeks after TBI. At 0,1, 3, 5, 7, 10, 14, 21 days after TBI, sensorimotor functions were assessed by neurological score and hanging wire tests. Body weight loss was also measured on each test day. Experimental data showed significant deficits in all tests from day 1 to day 7 after TBI. All deficits were recovered close to pre-injury baselines by day 21 after CCI. However, there were no statistically significant differences between Ngb-Tg and WT mice in all assessments during the 3-week TBI recovery period (Table 1). We also used Morris Water Maze to assess spatial memory acquisition at 15-21 days after TBI, but we did not find significant differences between Ngb-Tg and WT mice in latency for hidden and visible platform trials (Figure 3A), as well as in probe trials (Figure 3B). Lastly, we quantitatively examined traumatic brain lesion volume and found it was significantly reduced in Ngb-Tg mice compared to WT mice at 21 days after TBI (Figure 4).

**Table 1:** Sensorimotor Function Assessments

**Tests**	**Neurological score median (25th to 75th percentiles)**		**Hanging wire median (25th to 75th percentiles)**		**Body Weight, % of day 0**	
**Group**	**WT**	**Ngb-Tg**	**WT**	**Ngb-Tg**	**WT**	**Ngb-Tg**
Day 0	0	0	5.0 (4.8-5.0)	4.7 (4.9-5.0)	100	100
Day 1	3.0 (2.0-3.5)	5.0 (3.0-6.0)	3.0 (2.2-3.7)	3.0 (2.0-3.3)	97.2 ± 6.67	95.5 ± 3.74
Day 3	2.0 (1.0-2.5)	2.0 (1.0-3.5)	4.0 (2.7-4.3)	2.7 (2.2-4.2)	100.1 ± 6.02	97.5 ± 2.18
Day 5	1.0 (1.0-2.0)	2.0 (1.0-2.0)	4.0 (3.3-4.7)	4.0 (3.2-4.3)	99.7 ± 6.81	98.5 ± 2.94
Day 7	1.0 (1.0-2.0)	1.0 (1.0-1.0)	4.3 (4.2-4.7)	4.0 (3.2-4.1)	103.8 ± 6.96	97.8 ± 4.09
Day 10	1.0 (1.0-2.0)	0.0 (0.0-1.0)	4.3 (4.0-4.8)	4.0 (4.0-4.0)	103.8 ± 6.96	97.8 ± 4.09
Day 14	1.0 (1.0-1.0)	0.0 (0.0-1.0)	4.3 (4.0-4.8)	4.0 (3.8-4.0)	105.1 ± 6.74	99.4 ± 4.32
Day 21	1.0 (0.0-1.0)	1.0 (0.0-1.0)	4.3 (4.1-5.0)	4.0 (3.8-4.0)	108.3 ± 7.48	109.5 ± 4.36

Sensorimotor deficits and body weight loss were assessed before (day 0) and at days 1, 3, 5, 7, 10, 14, 21 after CCI. Results are expressed as median values and 25^th^ to 75^th^ percentiles for assessments of neurological score, hanging wire tests; and mean ± SD for assessments of body weight loss. N = 11 for Ngb-Tg and 15 for WT per group. In all assessments, there were no statistical significant differences between WT and Ngb-Tg groups.

In the past two decades neuroprotectants that seek to block or inhibit one specific step in the cascades of TBI have not been very clinically successful [24]. It has been shown that TBI triggers endogenous protective mechanisms that can prevent or limit brain damage. Methods that seek to augment the brain's own endogenous protection and repair signals may lead to new therapeutic strategies for stroke and related disorders [25]. Experimental studies from our lab and others have documented that Ngb is one of very few unique molecules for endogenous neuroprotection, which functions in stabilizing neuronal function and prosurvival genes under both normal rest and hypoxic/ischemic conditions, serving to protect against oxidative stress and preserve mitochondrial function [4,9,26]. Furthermore, investigators have recently been attempting to elucidate Ngb gene regulation mechanisms, identifying small molecules that can specifically up-regulate endogenous Ngb protein expression for the development of novel endogenous neuroprotection strategies for treating neurological disorders [19,27]. Thus in this study, for the first time, we tested whether Ngb overexpression has neuroprotective effects in TBI model of mice. Results from present work showed: (1) TBI significantly increased Ngb expression in peri-lesion of ipsilateral cortex at 6 h after TBI compared to sham in both WT and Ngb-Tg mice, but the increased Ngb level was significantly higher in Ngb-Tg mice than WT controls; (2) Ngb-Tg significantly reduced the levels of oxidative damage marker 3NT at 6 h after TBI in Ngb-Tg mice compared to WT controls; (3) Ngb-Tg mice exhibited smaller lesion volumes at 3 weeks after TBI compared to WT; (4) however, there were no significant differences in neurobehavioral deficits between the WT and Ngb-Tg mice after TBI for up to three weeks.

Ngb-Tg mice exhibited smaller cortical lesion volumes, suggesting that Ngb overexpression may prevent TBI-induced brain tissue damage. Oxidative stress has a mechanistic role in the pathophysiology of many neurologic diseases, including traumatic brain injury [15], and previous studies have shown that Ngb over-expression may reduce hypoxia/ischemia-induced oxidative stress in cultured neurons and a focal stroke mouse model [4,5,9]. Additionally, 3NT production at early time points after brain injury is associated with the degrees of TBI-induced oxidative tissue damage [15,28,29]. Thus, we tested and compared 3-NT production levels in the brain tissues of Ngb-Tg mice and WT mice after TBI. The significant reduction of 3-NT production in Ngb-Tg mouse brains suggested that Ngb over-expression may protect TBI brain damage, at least partially, by decreasing oxidative stress, which has been similarly observed in other studies of cells cultures and animal disease models [5,9,19]. Although we did know exactly why there was no effects of Ngb-Tg in oxidative stress reduction in the sham mice, but we may speculate that Ngb may have multiple functions for cell protection by maintaining hemostasis via stress sensing, or transducing signals or direct action. Ngb might respond stress to eliminate excessively elevated oxidative signaling and tissue damage, but is not or less effectively taking action under physiological resting conditions [4,8-11]. Therefore the basal level of 3NT in sham group was not significantly altered as observed in present work.

We acknowledge that the early reduction of 3NT production at 6 hours after TBI in Ngb-Tg mice might be correlated with the lesion reduction at 3 weeks, but other factors likely also contribute to the neuroprotection of Ngb-Tg in TBI. A number of laboratories, including ours, have demonstrated that Ngb is protective against hypoxic/ischemic brain injury. Although the underlying mechanisms remain poorly defined, initial evidence suggests that the neuroprotective effect of Ngb may be linked largely to the structural features related to O_2_ and NO binding. Furthermore, putative signal transduction and mitochondrial function preservation also may be involved in the protective mechanisms [4,8-11]. However the involved molecular mechanisms need to be investigated in further studies.

It is worthy of noting that, there are two published studies closely related to the present work. A recent study showed that over-expression of Ngb in cultured neurons was neuroprotective against mechanical injury in vitro. This study also showed increased Ngb expression levels with peak time of 6 hours after TBI, and these increased levels correlated to the severity of TBI in rats [23]. Thus, we picked only the time point of 6 hours after TBI for examining changes of Ngb expression and 3NT production after TBI in present study. One of our previous studies in focal stroke showed oxidative tissue damage biomarker malondialdehyde levels in ischemic hemispheres of Ngb-Tg were significantly reduced compared with wild-type controls at 8 hours and 22 hours after transient focal cerebral ischemia. Brain infarction volumes 1 day and 14 days after stroke were significantly reduced in Ngb-Tg mice. However, there were no significant detectable improvements in sensorimotor deficits for up to 14 days after stroke in Ngb-Tg mice compared with wild-type controls [5], which was similarly observed in present work in TBI mice.

We are aware that there are a few limitations in this study. First, the total Ngb protein expression levels in our Ngb-Tg mouse line is about 1.5 fold of WT control, which is relatively lower than when the Ngb-Tg mouse line was newly generated 5 years ago [5]. The decline of the Ngb protein level in Ngb-Tg mice may be mainly due to the endogenous depletion of inserted exogenous DNA fragments or inactivation of its promoter [30,31]. However findings from present work are still very informative and suggest that Ngb over-expression might be a new target for TBI therapy strategy development. We are working on a generation of neuron-specific and inducible Ngb-Tg mouse line for future studies of Ngb over-expression effects in TBI neurological outcomes and underlying molecular mechanisms. The second limitation is related to mechanisms. We only detected the reduction of 3-NT production in the Ngb-Tg mouse brains at the early time of 6 hours after TBI, suggesting there might be an antioxidative role for Ngb in vivo. However, for in vivo TBI models, it is difficult to unequivocally prove causality because reduced tissue damage may secondarily contribute to decreased oxidative stress and/or mitochondrial dysfunction. The third limitation is that we failed to detect significant differences in sensorimotor function or spatial memory function recovery between WT and Ngb-Tg mice. In part, this is because the TBI setting in our study might not be severe enough to cause long-term sensorimotor deficits since most of the deficits recovered close to the pre-injury baseline from one to two weeks after TBI. Also, the Ngb protein level in Ngb-Tg mouse brain might not be high enough. Alternatively, we may have simply lacked power and larger numbers of test animals would have helped. Further investigation is warranted to carefully define the role of Ngb in both long-term sensorimotor and cognitive deficits after TBI.

## Conclusions

These experimental results suggest that Ngb over-expression can reduce traumatic brain lesion volume, possibly through reduction of oxidative stress.

## Abbreviations

3NT = 3-nitrotyrosine; CCI = Controlled cortical impact; Ngb = Neuroglobin; Ngb-Tg = Neuroglobin over-expression transgenic; NSS = Neurological severity score; PBS = Phosphate buffered saline; TBI = Traumatic brain injury; TBS = Tris buffered saline; WT = Wild type.

## Authors’ contributions

SZ: Performed TBI model and immunohistochemistry, drafted manuscript. ZY: Performed behavior tests and draft manuscript. GZ: Helped in experimental design. CX: Performed western blots. KH: Performed immunohistochemistry. MJW: Helped in experimental design and performed TBI model. JML: Performed behavior tests and data analysis. EHL: Helped in experimental design and troubleshooting. XW: Designed experiments, analyzed data, drafted and finalized manuscript. All authors read and approved the final manuscript.
